# Study on the characteristic mechanisms of infrasonic precursors during the damage process of impending earthquake sources

**DOI:** 10.1371/journal.pone.0257345

**Published:** 2021-10-01

**Authors:** Wei Wang, Xiaorong Xue, Weisheng Chen, Xiaoyan Xue

**Affiliations:** 1 School of Information Engineering, Institute of Disaster Prevention, Beijing, China; 2 School of Electronic and Information Engineering, Liaoning University of Technology, Jinzhou, China; 3 Institute of Seismology, Beijing University of Technology, Beijing, China; 4 Beijing University of Technology, Beijing, China; Sapienza University of Rome: Universita degli Studi di Roma La Sapienza, ITALY

## Abstract

Infrasonic signals measured before an earthquake carry information about the size and development speed of the source fracture, the stress at the fracture site and the elastic properties of the geologic medium. The infrasonic signal has a stable time scale, and compared with other precursors, infrasound has a unique sensitivity to earthquake disasters. However, to date, there has been no relevant theoretical research on the mechanism of infrasonic anomalies, and information on the development of fracture sources cannot be obtained from these characteristics, which makes the application of this anomaly in earthquake prediction challenging. In this study, we obtained the characteristics of short-term and impending infrasonic anomalies based on the infrasound data of more than 100 strong earthquakes. With a range of elastic medium models with a large number of fractures, we completed the theoretical simulation of the formation process of infrasonic precursors during the formation of the main fractures, analyzed the physical evolution of acoustic signals when cracks are generated, and quantitatively described the stages of large fracture formation caused by the initiation and propagation of seismic cracks. Specifically, this study revealed the causes of various and complex forms of infrasonic precursors near the critical point and the causes of the time- and space-dependent characteristics of these precursors, such as a noticeable attenuation of the pulse number, a low frequency and a large amplitude, which verified the effectiveness of infrasonic anomalies as strong earthquake precursors.

## Introduction

The interpretation of acoustic precursor characteristics is the basis of the understanding of the main failure mechanism of earthquakes or rock fractures. Within the scope of studies on failure mechanisms and characteristics, such interpretations have been widely used to evaluate the integrity of rock masses and analyze the stability of geomechanical structures in the mining industry [1,2]. With the further development of monitoring technologies, earthquakes have been found to be the cause of infrasound [3,4]. Dickey reported on a unique set of infrasound observations of the Circleville earthquake that occurred on January 3, 2011; these observations were recorded by nine infrasound arrays in Utah [5]. Cansi [6,7] studied 136 earthquakes and obtained standard information on infrasound phase detection and earthquake location. Senkevich [8] proposed an experimental method to evaluate the seismicity of the Kamchatka area based on the analysis of geoacoustic signals. Infrasound disturbance is of major interest in research regarding earthquake prediction and can be used as one of the prediction factors [9]. Li et al. [10] applied infrasound in earthquake prediction, and the results supported the use of infrasound as an important and effective method for impending earthquake prediction.

Under the action of forces, the development of one or more primary cracks normally leads to rupturing. However, the initiation and growth of the primary cracks constitute only a certain phase of the crack evolution that determines the total rupture magnitude within the entire volume of a loaded sample. Nagao [11] constructed a Green’s function from the related convolution of focal earthquake sources and an Earth model and simulated the infrasound generated by multiple point sources; they proposed that infrasound data, such as seismic data, contain sufficient precursory information. As an associated phenomenon in the process of rock deformation and failure, acoustic signals are an effective information carrier and reflect the change in the internal structure of the rock. The detection of damage types ranging from cracks and corroded surfaces to large and complex structures by analyzing the critical acoustic emission (AE) parameters of rock damage has a high reliability [12]. Anomalous infrasonic waves are also one type of AE event. For monitoring, anomalous infrasonic waves have advantages of a low attenuation and long propagation, and their characteristics can be described by their amplitudes, shapes and frequency. According to Zhang and Li [13], for typical intraplate earthquakes, the plate interior can be regarded as a relatively complete rock block. In such a simulation experiment, the earthquake process is regarded as the damage and fracture process of the whole rock, and the block does not contain structural planes with obvious seismic structure characteristics such as faults; the only assumption regarding this block is that the strength of the seismogenic body is lower than that of the surrounding medium but that its stiffness is slightly higher (i.e., more brittle) [14]. Therefore, research on the loading of integral rocks is consistent with the changes in the mechanical properties of the medium of the geological body during loading and expansion. Additionally, such studies can also support research on the infrasonic precursors of earthquakes.

As the release of elastic waves from the rock interior is directly related to the generation of microcracks (damage), large-scale high-power AE events can often be monitored when macrorupture events are initiated [15]. The AE of rock samples is related to damage accumulation [16]. To obtain more information regarding the rupture process, numerous experiments on acoustic information have been carried out. Zhang and Zhou [17] utilized the integrated acoustic monitoring technique to detect the development of and energy evolution during unconfined failure in flawed granite specimens. Naoi [18] observed in an experiment that after the peak pressure, fracturing occurred, which was accompanied by a large seismic wave that was radiated by the dynamic propagation of the fracture; they also detected that the acoustic signal gradually moved along the fracture within the active area, with gradually enhanced intensity. In the process of cyclic loading and unloading, there is a certain relationship between the acoustic velocity and its associated characteristics and damage. Cyclic loading leads to the initiation of new cracks and the propagation of existing cracks; the existing cracks are connected in the most readily deformed horizontal direction, which increases the amplitude of the cyclic loading, aggravating the damage degree of the sample [19,20]. In a natural state, fractured rock masses are usually subjected to the original rock stress and its disturbance stress; however, sample damage is positively correlated with the absorbed energy, and the peak count of AE events increases with the increase in the number of loading cycles [21], revealing the quantitative mechanism of crack initiation, the periodic development of fracture development and the synchronous nature of the infrasound phenomenon.

There is a strong relationship between infrasound anomalous signals and earthquakes over time. However, the most fundamental problem is that the relationship between the fracture process and acoustic anomaly characteristics has not yet been established. At the same time, due to the lack of understanding of the irregularity, precursory complexity and nonlinear nature of geological bodies and methods to solve these problems, the infrasound information of geological body fractures cannot be qualitatively or quantitatively interpreted and analyzed, which limits the application of infrasound monitoring in the field of earthquakes. In addition, it is necessary to establish an appropriate physical model to reasonably explain such anomalous phenomena to further interpret the process of earthquake catastrophe and to provide a theoretical basis for assessing its effectiveness as a precursor. Currently, two models that interpret the development of precursor phenomena before an earthquake have been proposed: the expansion-dispersion model and the avalanche-type unsteady fracture formation model. Although these models differ greatly, they share the same presumption regarding the changes in the mechanical characteristics of the medium during the loading and expansion process. Based on this mechanical presumption, the current study established the quantitative relationships among the features of a medium with multiple cracks, the load and the precursory signals.

## Main characteristics of the anomalous infrasound signals of impending strong earthquakes

Exploring and identifying the precursory phenomena for earthquake preparation is a modern method in strong earthquake forecasting. Anomalous infrasonic waves observed in earthquake-prone areas present different features. Therefore, based on these features and previously gained experience, studies on this topic can be conducted. However, most of the previous related studies have focused on the sequential relationships between anomalous phenomena and earthquakes, and studies based on long-term statistical analyses are rare. After years of observation, our research team (the earthquake research team of the Beijing University of Technology) has found that the forms and frequencies of infrasonic waves differ greatly and that the maximum amplitude of an infrasonic wave is not proportional to either the earthquake magnitude or the distance from the epicenter to the observation site, which highlights the complexity of infrasound [22].

Based on these findings, we summarized the anomalous infrasonic features before a strong earthquake as follows.

### Relatively frequent abnormalities before earthquakes

**Fig 1:**
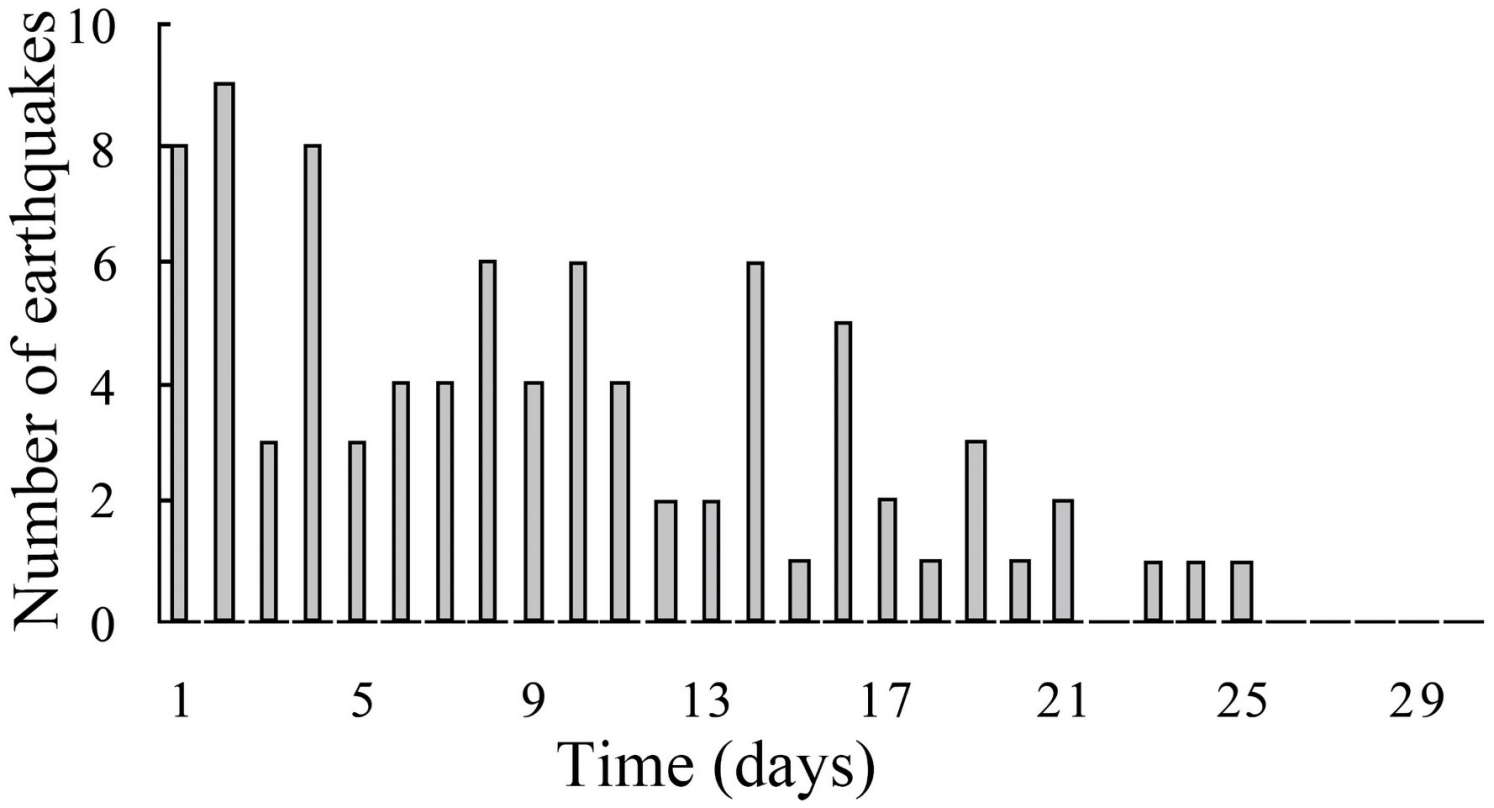
Time distribution of the anomalous infrasound signals preceding the earthquakes during 2002–2009.

Anomalous infrasonic signals were observed before 101 earthquakes with a magnitude (M) ≥ 7.0 worldwide from 2002 to 2009 (S1 File), and a total of 314 infrasonic anomalous signals occurred before these earthquakes were received. As shown in Fig 1, the number of earthquakes producing anomalous signals within 2 days before the earthquake is the highest, followed by between 1 and 4 days before the earthquake, and in 75.6% of the earthquakes (n = 78), signals were received 1–9 days before.

### Rapid increase in the amplitude of the anomalous infrasound signal before earthquakes

**Fig 2:**
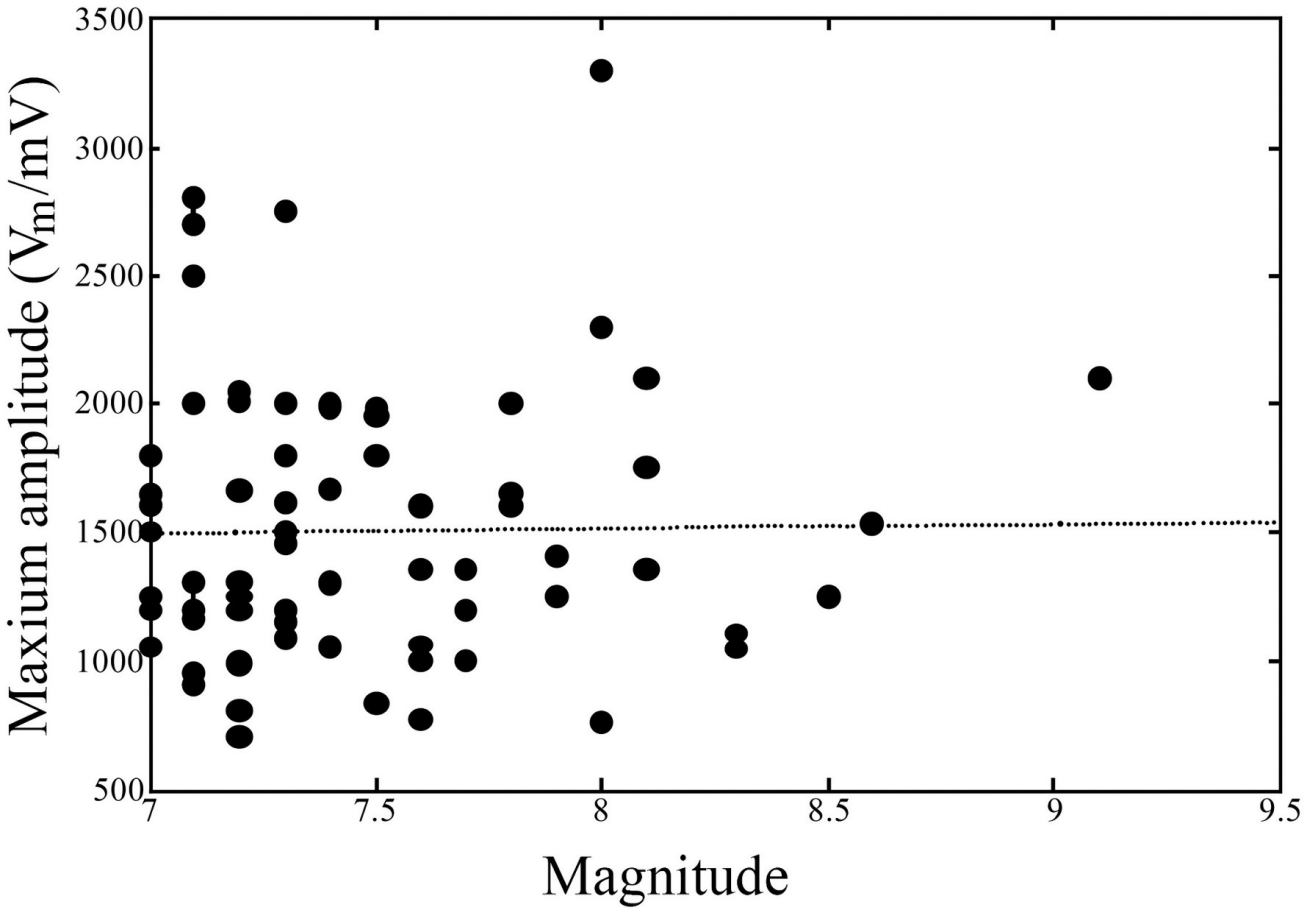
The maximum amplitude of the anomalous infrasound signal vs. the associated earthquake magnitude during 2002–2008.

In this study, we analyzed earthquakes with magnitudes between 7.0 and 8.0. According to the magnitude of each earthquake, we calculated the accumulated value and anomalous rate of all the seismic signals within 15 days before earthquake occurrence, and seismic signals occurring after interference signals were excluded. The results are shown in Fig 2.

The anomalous amplitude of earthquakes with M ≥ 7 is more than 1000 MV and that of earthquakes with M ≥ 8 is more than 2000 MV. The amplitude or energy of an infrasound anomalous signal is related to the magnitude of the corresponding earthquake. By analyzing the relationships among the magnitude, maximum amplitude and distance, it can be shown that the relationships between magnitude and maximum amplitude and between maximum amplitude and distance are not simple or directly positively correlated. Amplitude Vm is not proportional to magnitude M and distance D. There are examples of cases where Vm is relatively large when M is small and when Vm is relatively small when D is large. These results show the complexity of the relationships of Vm with M and D; therefore, it is necessary to study the mechanisms underlying infrasound generation and propagation.

### Diversity of anomalous morphologies

Based on more than 20 years of observation of and research on infrasonic anomalous signals, we found that infrasonic signals can be divided into three categories, and the characteristics of these categories were analyzed by evaluating the typical signals of the following three areas.

**Fig 3:**
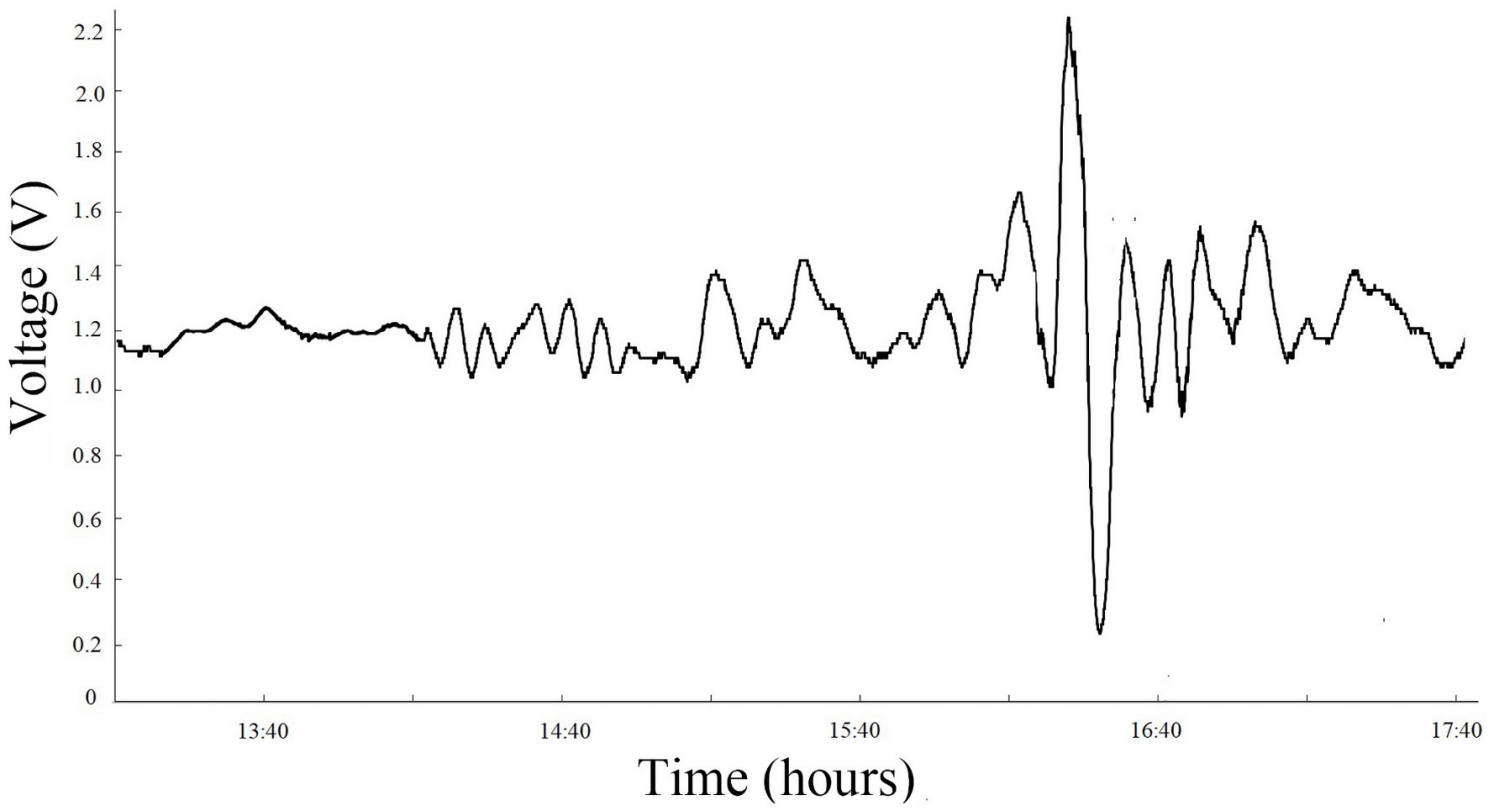
Anomalous infrasound signals on September 18, 1999.

**Fig 4:**
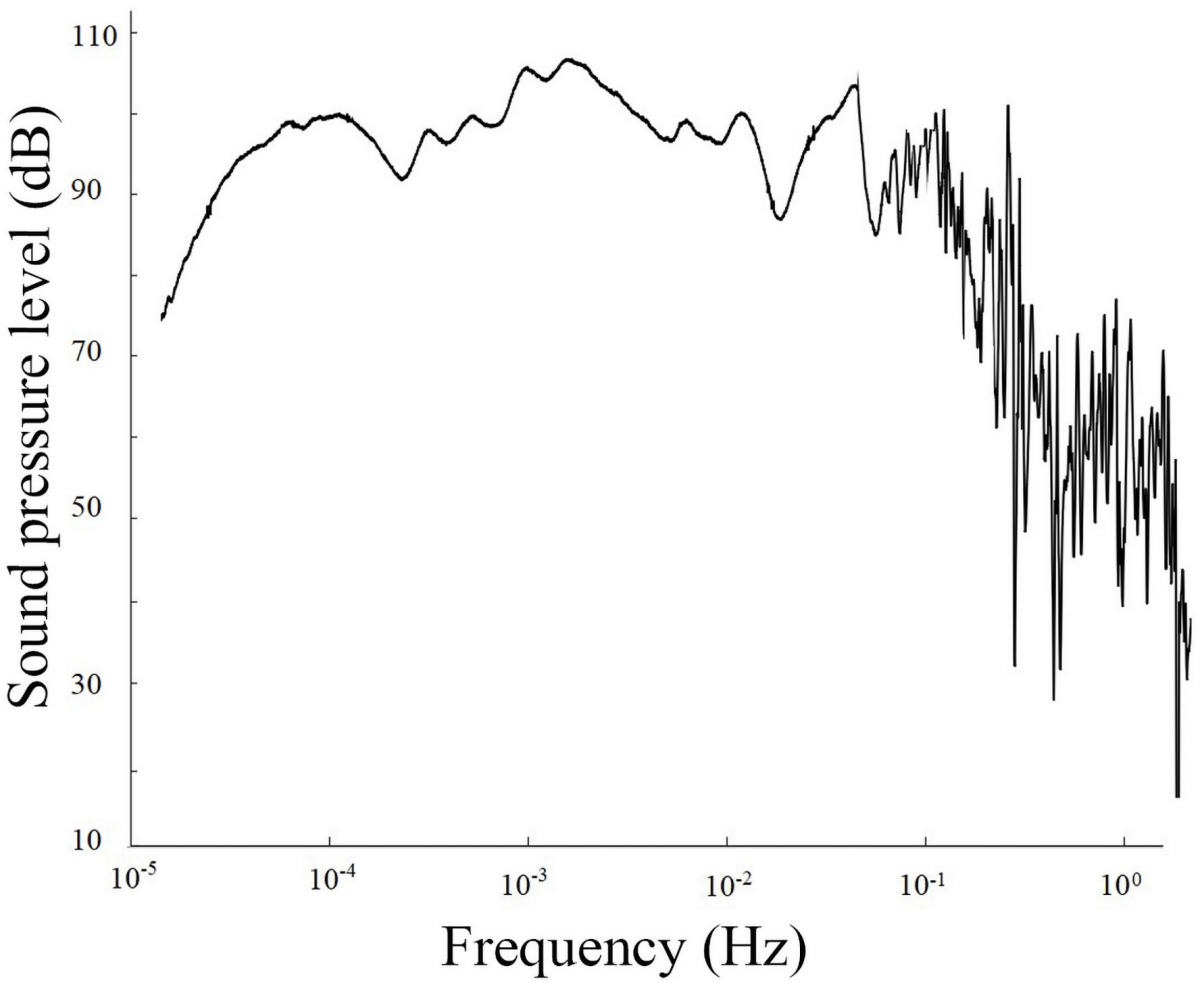
Frequency of anomalous infrasound signals on September 18, 1999.

1) On September 21, 1999, an earthquake with a magnitude of 7.6 occurred in Jiji, Taiwan. An anomalous infrasound signal was received on September 18, three days before the earthquake (Fig 3 and S2 File). This period was 14:40–18:40, and the sampling rate Fs was 5 s. This earthquake was subject to the third category of anomalous infrasound signal, and its cycle length reached more than 300 s. The spectrum analysis distribution of the signals is shown in Fig 4 (S3 File).

The signal shown in Fig 3 lasted for more than 1 hour, with a measured anomalous amplitude of 1.68 V, a simple waveform and a high signal-to-noise ratio. A noticeably large pulse signal was observed between 16:19 and 16:25. Fig 4 shows that the signal has multiple peaks between 0.0002 and 0.022 Hz, with a peak frequency of approximately 0.0044 Hz and the maximum sound pressure level of 103 dB.

**Fig 5:**
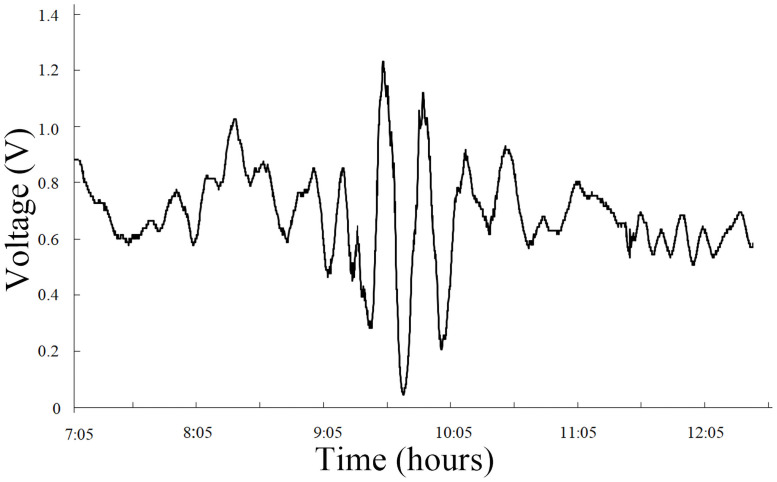
Anomalous infrasound signals on March 29, 2002.

**Fig 6:**
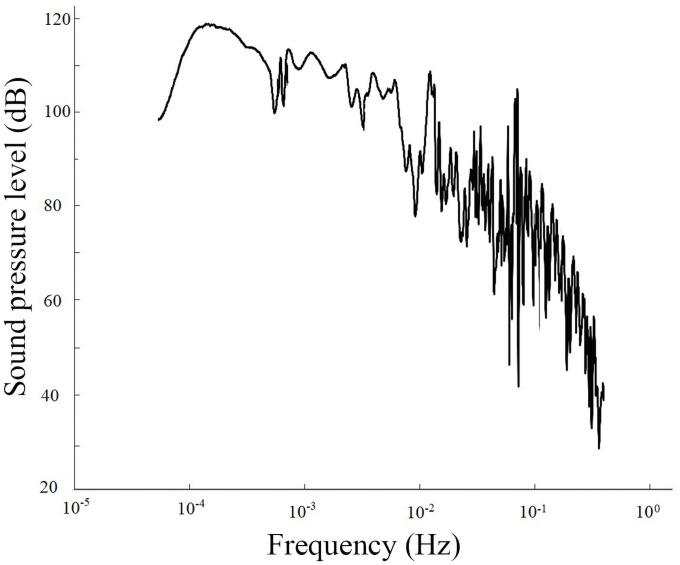
Frequency of anomalous infrasound signals on March 29, 2002.

2) On March 31, 2002, an earthquake with a magnitude of 7.5 occurred in the middle of the East China Sea and propagated toward Taiwan (Fig 5 and S4 File). The corresponding time interval was 8:05–11:40, and the waveform was relatively complex. The anomalous infrasound signal received corresponds to the second category. The spectrum analysis of the signals on March 29, two days before the earthquake, is shown in Fig 6 (S5 File).

The signal in Fig 5 lasted for more than 3 hours, and the anomalous amplitude was 1.81 V. Overall, the voltage amplitude increased, which was followed by a gradual decrease. Fig 6 shows that the signal exhibits multiple peaks between 0.0002 and 0.018 Hz, i.e., multiple dominant frequencies arise simultaneously. The peak frequency is approximately 0.0006 Hz, and the maximum sound pressure level is 114 dB.

**Fig 7:**
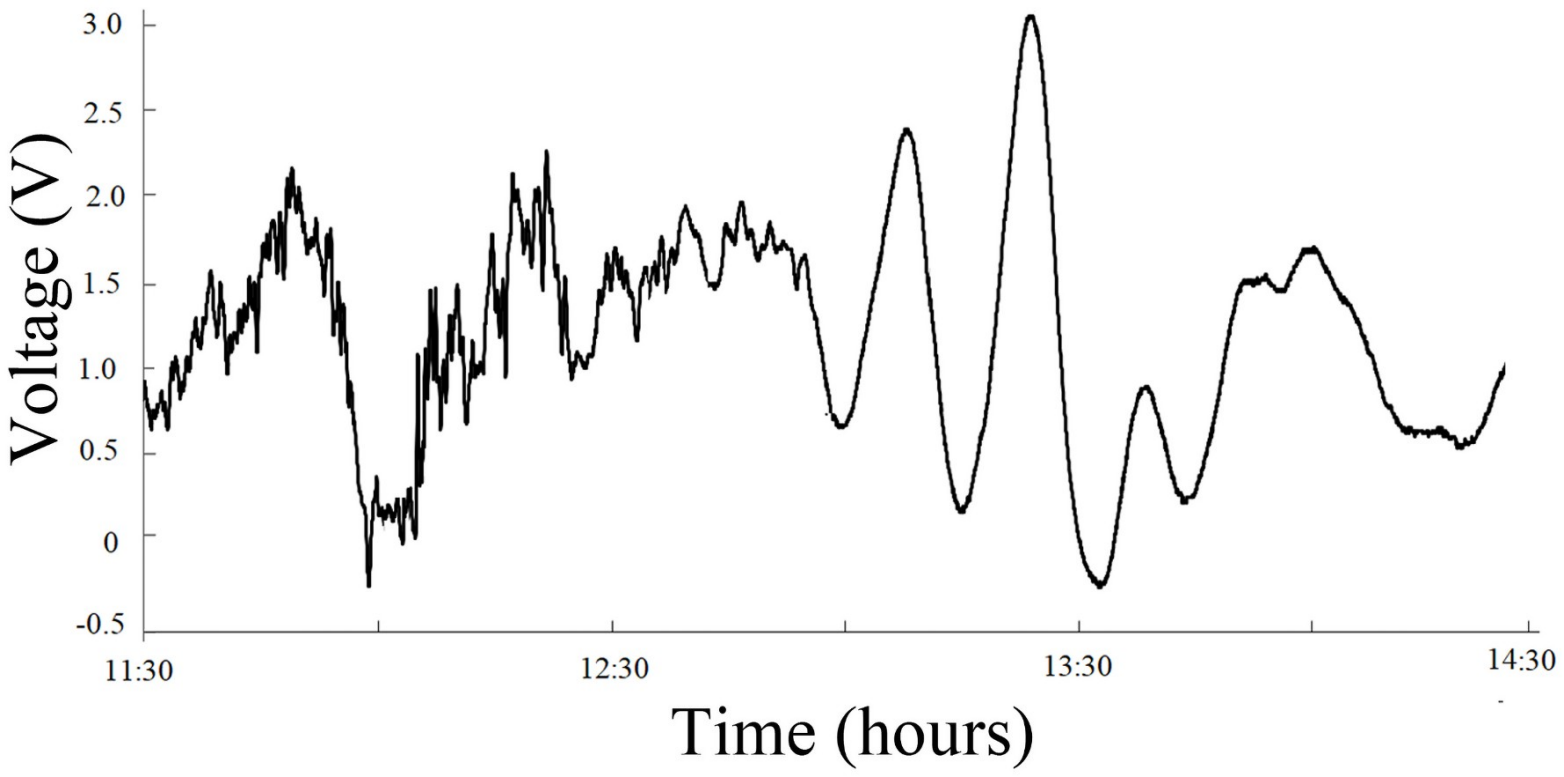
Anomalous infrasound signals on April 11, 2010.

**Fig 8:**
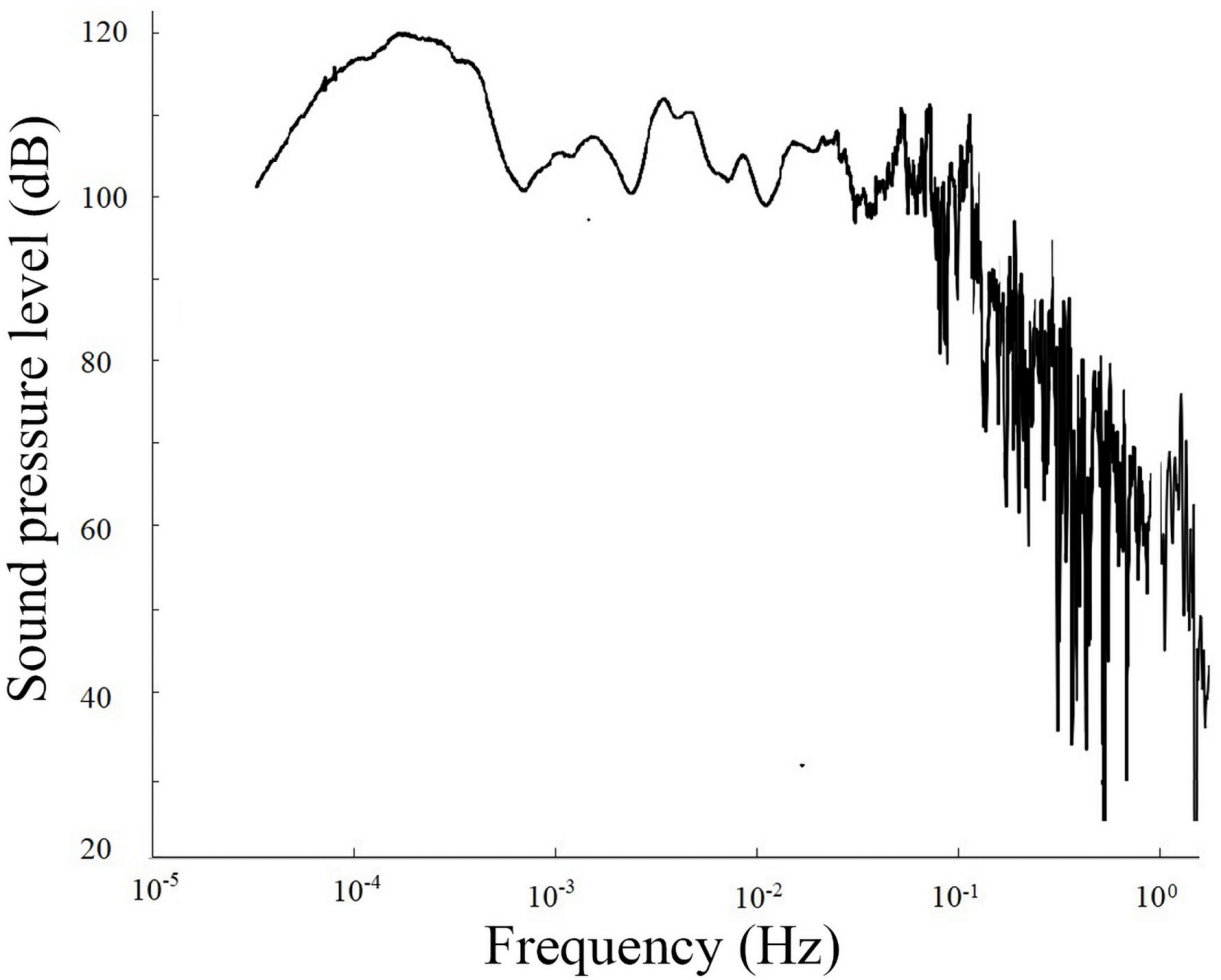
Frequency of anomalous infrasound signals on April 11, 2010.

3) On April 14, 2010, Yushu, Qinghai Province, experienced a shallow earthquake with a magnitude of 7.1 at a depth of 33 km. The infrasound anomalous signal was received on April 11, three days before the earthquake (Fig 7 and S6 File). The corresponding time interval was 11:30–13:30, and the sampling rate Fs was 1 s. The spectrum analysis of the signals is shown in Fig 8 (S7 File).

The signal diagrams and time-frequency distributions of the above three types of signals show that the signal types can be described as follows. The first type of signal has a smooth waveform and a single frequency, and the amplitudes in the positive and negative directions are approximately the same; the waves form one group or multiple groups, and the number of waves in each group is generally 2–7. The second type of signal consists of several dominant-frequency waves, with complex waveforms and effective signal durations reaching as long as tens of thousands of seconds. The third type of signal is a one-way pulse signal, with a duration of approximately thousands of seconds and a cycle of approximately 1000 s.

Based on the abovementioned analysis, apart from their characteristics of sudden and rapid changes, the infrasonic anomalies are also complex and diverse in morphology, and the time at which the maximum anomalous amplitude appears corresponds to the point of highest energy (as shown in Figs 3, 5 and 7); additionally, the dominant frequency is low and appears at approximately 10^−3^ Hz (as shown in Figs 4, 6 and 8). Is the obvious increase in the amplitude a reflection of the instability and failure stage, and how does the dominant frequency of 10^−3^ Hz come into being? To answer these questions, research on the characteristics of earthquake precursors must consider the mechanism of earthquake event preparation.

## Infrasound characteristics of impending earthquake rupture

### Rupture process and numerical simulation

Earthquake occurrence is a process of damage accumulation induced by fracture damage evolution in geological bodies. In fact, the nonlinear characteristics (pre-earthquake displacement, rock weakening in the source area and fault creeping) of impending earthquakes mainly result from microcrack damage accumulation over time. The characteristics of a medium in a seismogenic area are the basis of earthquake preparation and precursor monitoring. Before an earthquake, the affected geological body undergoes the formation of a rapidly increasing number of fissures, then the slow expansion of fractures, and finally the rapid dislocation of those fracture during the earthquake. Crack initiation and propagation occurs near the tips of the existing cracks, leading to the main failure; the characteristics of infrasonic abnormalities are closely associated with the eventual formation of macrocracks due to the damage accumulation in the geological body [23].

To elucidate the nature of the precursory phenomena, laboratory experiments on the mechanical properties of rock before rupture have been widely adopted by scholars. A commonly used experimental method is to place the sample in a high-pressure chamber [24,25] and apply uniaxial compression. During compression, the changes in the elastic wave velocity of the sample and the volumetric increase in the sample (i.e., expansion) are continuously recorded (particularly, the expansion of the sample is of significance for the interpretation of the trends of the surface motion near the earthquake source). In the current study, the changes in the propagation velocity of the stress wave and in the volumetric strain were investigated with a model of a fractured medium under the conditions of uniform compression and plane strain.

For the current task, the medium considered here expands; that is, during loading, the changes in its volumetric strain are not linear, and even under the condition of pure shearing, and can never reduce to zero. This assumption is adopted because there is only one elastic symmetry plane in the equivalent medium, and the direction of most cracks that are formed under uniaxial compression is close to that of the compressive strain.

To associate the density of the microcracks with the parameters of the material, we considered the material to be in a metastable state before rupturing. The transition from a metastable state to a stable state is realized through the generation of new microcrack accumulation. When the density of the microcracks among the crack group reaches a certain limit value, i.e., the formation of one additional microcrack will cause the instability of the entire crack group, this state is considered metastable. Our study focused on the accumulation of microcracks. The crack surface attenuates energy, which leads to the reclosing of the microcracks. When the density of the original microcracks reaches a certain threshold, the interaction and transfer of forces will occur among the crack group, which leads to the presence of larger cracks, resulting in larger-scale ruptures.

For any rock stratum, the cracks determine the earthquake source area as well as the deformation around the source area. For a deep fractured rock with many cracks, it is reasonable to assume that the two sides of the crack are closed and that they interact with each other in accordance with the friction law. As the relative sliding of the two sides is restricted by the pressure value along the normal direction of the crack, shear strain should be accompanied by volumetric changes. In addition, the medium containing the crack is considered an analog of the material experiencing internal embrittlement.

According to the theory of crack formation, the number of initial cracks per unit volume per unit time under standard conditions is determined by the following formula:

$$I=z\beta N\text{exp}\left(-\Delta \text{G/}\kappa \text{T}\right)$$ (1)

where *Z* is the Zelidovich factor, *β* is the critical initial growth rate, *N* is the number of growth centers per unit volume, and ΔG is the power under which the critical initial crack is formed. The numerical form of *Z*, *β*, *N* and ΔG depends on the specific dynamic characteristics of the studied system; however, the interdependence among these parameters is the same in all cases. Compared with the exponential factor (the change in the exponential factor is neglected), the density of random nodes is obtained as follows:

$$x=consta^{3}\text{exp}\left(-\Delta \text{G/}\kappa \text{T}\right)$$ (2)

where α represents the critical scale. The duration of sample loading, where θ = *t*_*k*_, depends on the conditions *x* = *x*_k_ for the critical density under constant external parameters (stress σ and temperature *T*).

Only microcracks that satisfy the condition of *r* >α are considered stable. These stable microcracks constitute the random nodes of the infiltration model. At this moment, the duration time *θ* = *t*_*k*_ is just the time required for the transition from microcracks to larger cracks.

To evaluate the effect of the external load on the formation of microcracks, Δ*G* can be written as follows:

ΔG=F−V−A (3)

where F represents the change in the surface energy, *V* represents the elastic energy stored in microcracks, and *A* represents the power exerted by the external force. Based on dimensionality, *F* = *dr*^*2*^, *V* = *br*^*3*^, and *A* = *Cr*^*3*^σ, where σ is the pressure and *b*, *c* and *d* are constants.

Based on ∂Δ G/ ∂*r* = 0, the following equations can be obtained:

$$\Delta \text{G}\left(\alpha \right)=4d^{3}/27{\left(\text{b}+\text{c}\sigma \right)}^{2}$$ (4)

$$\alpha =2d/27\left(\text{b}+\text{c}\sigma \right)$$ (5)

Then, *θ* can be calculated as follows:

$$\theta =consta{\left(\text{b}+\text{c}\sigma \right)}^{3}\text{exp}\left[4d^{3}/27{\left(\text{b}+\text{c}\sigma \right)}^{2}\right]$$ (6)

When σ varies within a large range, the relation between lg*θ* and *θ* will deviate from the linear relation, exhibiting a hyperbolic form. When σ varies within a small range, however, the relationship described in Eq (6) has no difference from that described by *θ* ϒ exp[(*U* − *cσ*)/*kT*], i.e., the Zhurkov durability criterion.

Therefore, the assumption of a metastable state agrees with the law determined by experiments. From this point of view, the density set *x* can be linked to the measured *θ* value. For instance, *x*/*x*_*k*_ = *t*/*θ*, where *t* is the observation time, and this relation also supports the conclusion that the percolation model is comparable to the experimental data of the AM of rock failure.

Near the critical concentration value, the total number of pulses in the first-order approximation can be compared to the correlation radius of percolation theory, and the total number of acoustic pulses is proportional to a certain crack length power:

$$R=const{\left(x_{k}-x\right)}^{-v}$$ (7)

where *V* is the critical exponent, with a value of 0.9 under a three-dimensional condition. In the absence of detailed information on the fracture micro process, the applicability of Eq (7) can be tested according to its asymptotic properties and critical values.

The morphology of the total number of AE pulses when the stress σ approaches the critical range is modeled as follows:

$$N=consy{\left[{\left(\frac{\sigma_{k}}{\sigma }\right)}^{2}-1+\Delta \right]}^{-v},\Delta =\alpha /L$$

where α is the average size of the microcracks and L indicates the linear features of the sizes of the samples. The introduction of the additive term Δ is based on the consideration of the limitation of the sample and the influence of its positioning stage. Within the *σ*→*σ*_*k*_ range, the graph of the total number of AE pulses and the stress σ on a double logarithmic scale exhibits a linear relationship, which indicates the consistency between theory and our experiment.

The actual value of the critical exponent (1–0.9; 2–1.6; 3–0.8; 4–1.0) is also highly consistent with the theoretical percolation value of 0.9. With the AE experimental data, we established an analogous relationship with the empirical criterion of seismology, i.e., the dependence of the number of earthquakes on the energy or magnitude.

If the phenomenon that the pulse amplitude recorded at the time of fracture formation is proportional to the size of the fracture is observed, the criterion can be directly derived from the percolation mechanism.

According to the percolation model, when *x* = *x*_*k*_, the number of fractures is distributed according to *n*_*s*_ ϒ *s*^*-t*^, where *n*_*s*_ is the number of node sets composed of *s* nodes, and t is the critical exponent, which is assumed to be 2.5 under a three-dimensional condition. Below the critical density range, the node sets display a more complex relationship. For the Liejie network, the node set parameters are calculated as follows:

$$n_{s}=\frac{1}{\sqrt{2\pi }}\chi s^{-5/2}\text{exp}\left[-\frac{s}{2}{\left(\frac{x_{k}-x}{x_{k}}\right)}^{2}\right]$$ (8)

or

$$\text{ln}n_{s}=A-\gamma \text{ln}s$$

where $\gamma =\left[\frac{5}{2}+\frac{s}{2\text{ln}s}{\left(\frac{x_{k}-x}{x_{k}}\right)}^{2}\right]$.

When the density is close to the critical value (*x*→*x*_*k*_), the slope γ of the repetition rate graph will decrease. This characteristic of parameter γ has been observed in both AE laboratory research and mine experiments [21].

### Simulation and interpretation of short-term and impending precursory anomalies

The model described above is based on the general assumption of critical phenomena, i.e., the main effect of the fracture process is the wide fluctuation in material parameters. The percolation model describes the initial stage of the fracture process and can obtain a quantitative estimation of the threshold point and the critical exponent value, which can be compared with the observed value. After the transition from the percolation problem of the nodes without interaction to a more complex statistical problem, more realistic descriptions of the fracture process can be obtained. This process takes into account the metastable changes caused by the development of large-scale fractures and the disappearance of small fractures; additionally, the directional characteristics of the fracture and main fracture propagation in the external field can be obtained. Although this treatment can lead to a change in the density threshold and critical exponent, the overall process remains the same. However, to describe more complex dynamic phenomena, such as sound propagation, phenomenological critical phenomenon theory, such as Landau phase transition fluctuation theory, can be used to replace statistical theory based on the existing level of knowledge regarding the microscopic parameters of fractures.

Let a time-related series parameter be the probability of macroscopic fracture *η*. When *x*≤*x*_*k*_, *η* = 0; when *x*>*x*_*k*_, *η*>0. We next discuss the system described by the time-related series parameter *η* = *η*(t). Near the critical point, the characteristic time of the change in *η*(t) occurs earlier, and the remaining degree of freedom of the system just reaches equilibrium, which demonstrates that there is a thermodynamical potential energy *∅* that is dependent on *η*, and *∅* = (*P*, *T*, *η*).

Let the given inequality values representing the entire system be *η*≠ 0. According to the basic thermodynamic principle of irreversible processes, the change rate *Δη* is proportional to the conjugate force, and when it slightly deviates from equilibrium, the following can be obtained:

$$\frac{d\Delta \eta }{dt}=-\alpha \frac{\partial \phi }{\partial \eta }$$ (9)

where α represents the dynamic coefficient. Furthermore, when *Δη* deviates slightly from the equilibrium position *η*_0_ and the constant value of thermokinetic change, Eq (8) can be transformed into a linear equation describing the temporal eigenvalues of *t*_0_ and the relaxation of sequence parameter *η*:

$$\frac{d\Delta \eta }{dt}=-\frac{\Delta \eta }{\tau_{0}},\tau_{0}^{-1}=\alpha /\chi$$ (10)

In Landau’s theory, it is assumed that the dynamic coefficient *Q* is constant at the transition point and that the sensitivity x becomes infinitely large. Therefore, the following can be obtained:

$$\tau_{0}^{-1}=\alpha c\left(T\right)\cdot \left(P_{k}-P\right)$$ (11)

Around 2000, Carcione introduced the relaxation mechanism into the dissipative force of Biot’s two-phase theory and obtained the expression of dissipative force. The relationship between relaxation time and frequency can be determined according to the following equation [26,27]:

$$\tau_{\varepsilon C}=\frac{1}{2\pi f_{0}Q_{C}}\left[\sqrt{Q_{C}^{2}+1}+1\right]$$ (12)

$$\tau_{\sigma C}=\frac{1}{2\pi f_{0}Q_{C}}\left[\sqrt{Q_{C}^{2}+1}-1\right]$$ (13)

where *τ*_*εC*_ and *τ*_*σC*_ represent the strain relaxation and stress relaxation times under the relaxation dissipation mechanism, respectively.

Therefore, near the transition point, the relaxation time increases sharply, and the frequency *f*_0_ decreases. Near the critical point, equilibrium establishment is extremely slow, which leads to an obvious attenuation in the number of sound pulses and a reduction in the frequency. If there is sound wave propagation at a frequency of *ω* = 2*πf*_0_, i.e., periodic adiabatic compression and rarefaction, in the target medium, the wave propagation depends on the effective longitudinal modulus *M*(*iω*),

$$M^{-1}\left(\text{i}\omega \right)=M_{0}^{-1}+\frac{\alpha k^{2}}{A+i\omega }$$ (14)

where *A* = *τ*_*0*_^*−1*^ = *αc*(*T*) ϒ (*P*_*k*_-*P*), k = const, and ${\text{M}}_{0}=\rho \nu_{0}^{2}$.

The sound wave propagation velocity and absorption coefficient can be expressed as follows:

$$\text{v}={\text{v}}_{0}\left(1-\frac{M_{0}k^{2}}{A^{2}+\omega^{2}}\right),\beta =\frac{k^{2}}{2}\frac{\alpha \rho \nu \omega^{2}}{A^{2}+\omega^{2}}$$ (15)

The sound velocity decreases before the rupture, while the absorption coefficient of a signal with a low frequency decreases and the longitudinal modulus *M*(*iω*) increases. Because of its low frequency, the infrasound wave exhibits little attenuation. Therefore, it can propagate a long distance and can be detected far from its source [28].

### Simulation experiment and results

Starting from the above fracture process, we introduce a three-dimensional network within the selected material volume (its volume is large enough to avoid the influence of the boundary), with a pitch of *α*. The value *x*_0_ is specified for the density of microcracks or voids in the mesh. If the density of microcracks or a gap within the mesh is greater than or equal to *x*_0_, the mesh is considered open; otherwise, it is considered closed. Then, the volume of the material can be replaced by a network of open and closed meshes. To characterize the distribution of the open meshes, the scale distribution function of the adjacent open mesh and the average scale of all adjacent open nodes throughout the volume of the material are introduced. Next, the pitch of the network is increased *b* times, and the averaging program is repeated. According to the similarity of the macro- and microfracture processes, it is reasonable to assume that the distribution of structural defects (the scale distribution of all open adjacent open meshes, the average scale of all adjacent open meshes, etc.) remains unchanged at different scales of investigation.

The formation mechanisms of these structural defects at each scale may have their own characteristics. Therefore, the accumulation process of structural defects does not depend on the details of the interaction between adjacent elements in the material but rather on some large-scale parameters. To describe these parameters, the value of variable *x* is selected from the thermodynamical variables of the research system, and it is assumed that when *x* reaches its critical value, i.e., *x* = *x*_*k*_, fracturing begins. Therefore, the fracture process described above is validated when the condition (*x*_*k*_-*x*)/*x*_*k*_ ≤ 1 is satisfied.

If the density of the initial microcracks with the characteristic scale *α* (equivalent to the radius of interaction) is *x*, the critical density *x*_*k*_ corresponds to the density of macrocracks in the medium. For three-dimensional cases, the formula *x*_*k*_ = 0.338 can be given theoretically. In the 35 indoor fracture tests conducted by the earthquake research team of Beijing Industry University, certain parameters were held constant for all solids, i.e., *K* = *N*^-1/3^*L*^-1^, where *N* is the number of microcracks, with a scale of *L* unit volume. In these tests, the amount of *L* and *N* changed by 4 and 12 orders of magnitude, respectively, but *K* was held constant at 2.5–5. The theoretical value of *x*_*k*_ was used to express *K*, and *K* = 2.34 was obtained, which is in accordance with the experimental value obtained by our team.

TtTTo connect the density of microcracks with the parameters of the material, and the material before fracture is considered to be in a metastable state. The transition from a metastable state to a stable state is achieved by the accumulation of small new states. In this study, focus is placed on the accumulation of microcracks. The cracked surface influences the energy, which leads to the reclosure of microcracks on a small scale. From the critical scale *α*, only those microcracks for which *γ* >*α* are stable. These stable microcracks become random nodes in the percolation mode.

**Fig 9:**
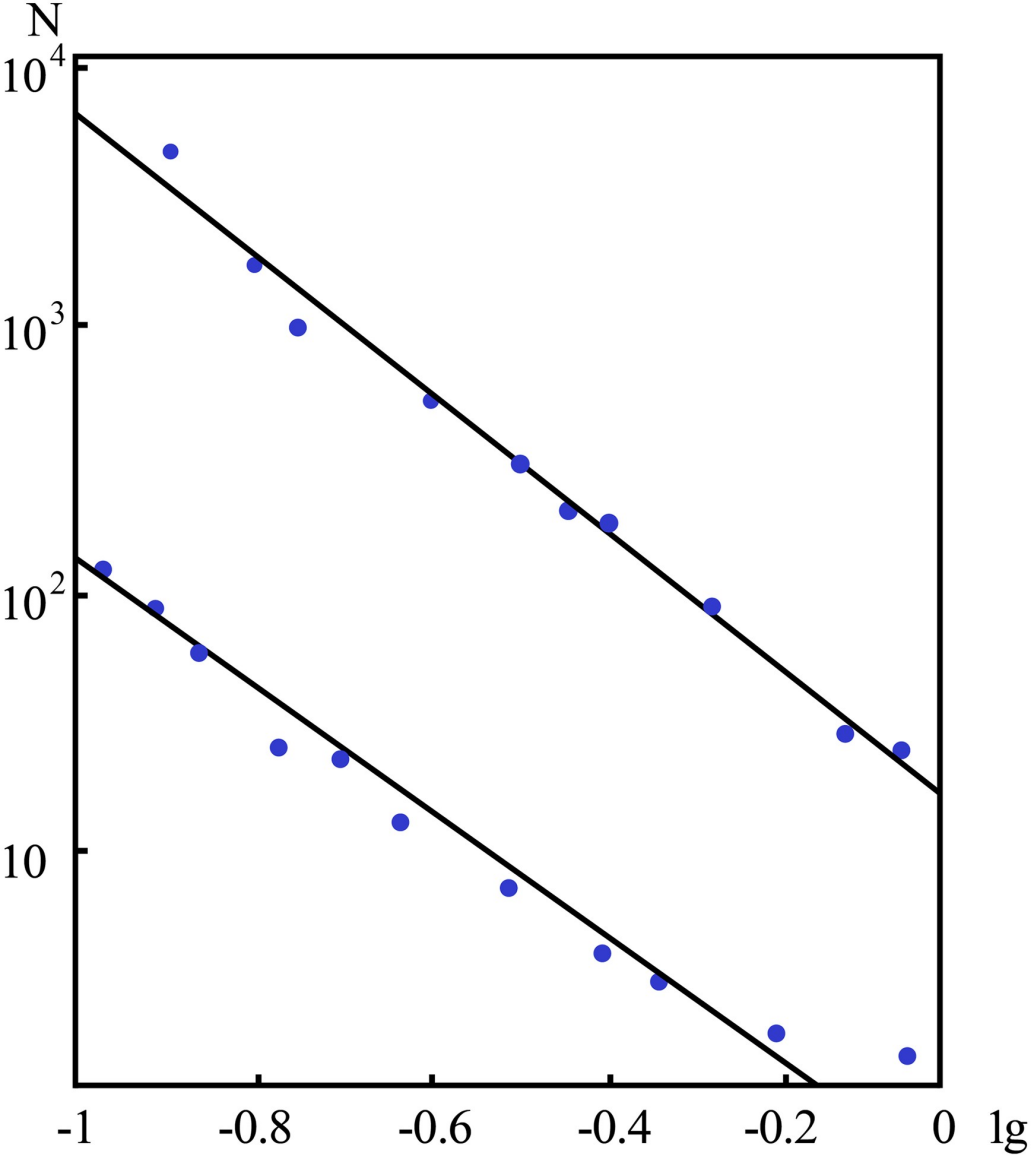
Characteristics of the total AE number *N* near the critical stress (representing the experimental values).

Fig 9 (S8 File) shows the experimental material obtained with granite samples via compression at a constant rate, and the calculated values with the total number *N* of AE pulses when the stress *σ* approaches the critical value are compared with the experimental data.

**Fig 10:**
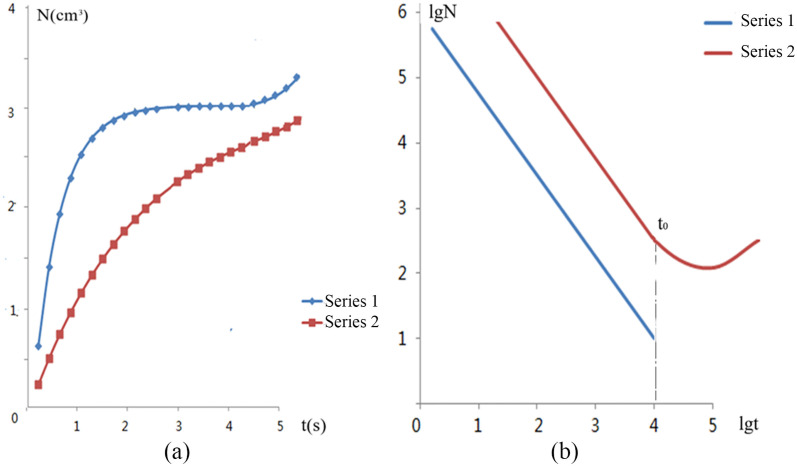
Granite compression experiment. (a) Fracture accumulation under uniaxial pressure. Curve 1, 1-*σ* = 85 MPa; Curve 2, 2-*σ* = 11 MPa. (b) Intersection of fracture formation velocity with time under normal pressure. Curve 1, 1-*σ* = 85 MPa; Curve 2, 2-*σ* = 11 MPa.

Fig 10 (S9 File) shows the fracture accumulation curves under two loads and the changes in the fracture velocity of granite material as an example. Fig 10(a) shows the fracture accumulation curves under the two loads. Curve 1 corresponds to a load of *α ≈* 0.7 as the rupture strength. Under this load, no effective source or development source is formed, and the process shows no more advancement than the volume accumulation process of microfractures. This process exhibits an attenuation trend, and with the coordinate lg *Ṅ* − lg *t*, it is expressed as a straight line throughout the entire time period (Fig 10(b)).

## Discussion

To explain the development of precursory phenomena before an earthquake, this study analyzed the anomalous features of infrasound based on the previous findings of our team, which were then associated with the regional seismogenesis and earthquake driving mechanisms.

The change in the functional relation of the fracture accumulation dynamics is evidence of a fracture source. In Fig 10(b), time *t*_0_ can be considered the starting point of short-term prediction because the development speed of the earthquake source increases exponentially. From *t*_0_ on, the parameter values increase rapidly, unstable development rapidly continues, and larger fractures appear or the existing cracks suddenly expand. This moment can be regarded as the indicator of the starting stage of unstable fracture source development.

Due to the complexity in handling the problem of the destruction of the geological body before an earthquake, numerical simulation of the earthquake preparation process is difficult to perform using a quantitative analysis method, except with a few simplified models. In this study, we described the dynamic phenomena of sound transmission based on Landau phase transition fluctuation theory. Our results showed that the sound anomaly produced at the transition point between the two stages of the fracture process has the characteristics of a high amplitude and a low frequency, and the attenuation is small, which can be monitored over a long distance. The calculated values are consistent with the observed values. Near the critical point, the relaxation time increases rapidly. The establishment of equilibrium is extremely slow, and the change speed under obvious strain and stress over a long period can be regarded as the precursory sign of a strong earthquake. This point leads to the noticeable attenuation of the number of sound pulses and the appearance of a low-frequency signal. Our simulation results verified that the generation of anomalous infrasound signals is associated with the accumulation of a large number of macrocracks. They form in the macrofracture stage, i.e., the final stage of the multiple processes during which cracks accumulate and then extend and become a network through interacting and coalescing. Our results are consistent with those reported in rock compression experiments [24], according to which the infrasound parameters abruptly change at the turning point of the fracture stage. Based on the analysis of the rock fracture process and mechanical performance under an axial force, Li et al. [29] found that the trend of the variation in AE from rock samples was basically consistent with the observed stages of the failure process.

In a cyclic stress experiment, Trippetta et al. [30] measured the elastic moduli of seismogenic Triassic evaporites and categorized the observed changes in the elastic moduli during the failure process into three stages. In addition, other rock parameters such as porosity affect the velocity of earthquake waves [31]. On the other hand, the stages of the failure process of geological bodies will cause changes in these parameters. In this study, we verified that the low-frequency and high-amplitude infrasonic anomalies had a stable time scale and may be used as an indicator of the beginning of the mechanical instability stage of failure.

## Conclusion

Currently, studies on earthquake infrasound primarily depend on simple assumptions regarding the earthquake source process and statistical descriptions of phenomena. Although these models explain some phenomena of earthquake precursors, they are subject to qualitative explanations and therefore cannot explain the complexity of earthquake precursors. In this study, for the first time, we associated infrasound with the genesis and development of earthquakes. Based on the consideration of stress and geological structure, we explored the characteristics of infrasound excited by the rupturing of internal rock damage. In addition, we established quantitative relationships among the characteristics of a geologic medium with multiple cracks, the loads and the precursory indictors and provided the mechanism controlling the infrasound characteristics.

The main contributions and conclusions of this study are as follows:

1. Based on the findings of previous research, we summarized the characteristics of anomalous infrasonic signals before strong earthquakes. The occurrence of infrasonic abnormalities has a stable time scale, the morphologies of the abnormalities are complex and variable, and the frequencies of the anomalous infrasonic waves are low, with a dominant frequency near 10^−3^ Hz.
2. We quantitatively explored the development process of the fracture source in an inhomogeneous system. Based on the mechanical method, the damage accumulation and expansion velocity of the fracture source were given. The fracture process includes two phases. From a certain time point, the parameters increase sharply, which can be used as an indicator of the initiation of the unstable development stage of the fracture source. This makes it possible to solve the problem of short-term prediction of earthquakes based on the quantitative study of the development dynamics of fracture sources.
3. Based on Landau’s theory, the association of the accumulation of a large volume of microcracks with the emergence of infrasonic signal abnormalities was evidenced. These microcracks emerge in the macroscopic fracture stage, that is, the final stage of the multistage process, due to crack accumulation, interaction and merging. Additionally, we described the amplitudes of the changes in infrasonic wave velocity and explained the reasons for the morphological complexity and low frequency of the waves, thereby explaining why infrasound can be monitored remotely. We also verified that infrasonic emission occurs before macrofracture formation and indicates the beginning of the mechanical instability stage. The results of this study suggest that infrasound monitoring is an important means of detecting the accumulation and expansion of fractures. It also serves as an important method for research on the dynamic development of the fracture source.

## Supporting information

S1 File Abnormal infrasound signals before earthquakes with a magnitude ≧ 7.0 worldwide during 2002–2009. (PDF)

S2 File Infrasound anomaly signals received 18 days before the Jiji m S7.6 earthquake in Taiwan on September 21, 1999. (XLS)

S3 File Spectrum of infrasound anomaly signal before Jiji earthquake in Taiwan. (XLSX)

S4 File Infrasound signals two days before the M 7.5 earthquake in the East China Sea over Taiwan on March 31, 2002. (XLS)

S5 File Spectrum analysis of pre earthquake signals of the Mid East China Sea earthquake in Taiwan on March 31, 2002. (XLS)

S6 File Infrasound anomaly signals received three days before the Yushu m7.1 shallow earthquake on April 14, 2010. (XLS)

S7 File Spectrum analysis of infrasound anomaly signal before Yushu earthquake in Qinghai on April 14, 2010. (XLSX)

S8 File Characteristics of the acoustic emission pulses (total number, N) near the critical stress state (the point represents the experimental value). (DOCX)

S9 File Data of crack accumulation and crack velocity in compression experiment of granite specimens. (XLSX)
