# Supplementary material for: Study on the characteristic mechanisms of infrasonic precursors during the damage process of impending earthquake sources
Source: PLoS One. 2021 Oct 1;16(10):e0257345. doi: 10.1371/journal.pone.0257345 (PMC8486096; doi:10.1371/journal.pone.0257345)
Supplement: S1 File — (PDF) [file pone.0257345.s001.pdf]

S1. File. Abnormal Infrasound Signals before earthquakes with a magnitude ≧ 7.0 worldwide during 2002-2009

Serial no.	Date	Magnitude	Longitude and latitude	Depth (km)	Date of abnormalities (d)	Maximum amplitude (mV)
1	2002-01-03	7.2	17.6°S, 167.9°E	21	None	
2	2002-03-03	7.4	36.5°N, 70.5°E	225	2/20, D-11	1980
3	2002-03-06	7.5	6.0°N, 124.2°E	31	2/20, D-14	1980
4	2002-03-31	7.1	24.3°N, 122.2°E	32	3/19, D-12	1060
5	2002-04-27	7.1	13.1°N, 144.6°E	85	4/23, D-4	1630
6	2002-06-28	7.3	43.8°N, 130.7°E	566.2	None	
7	2002-08-19	7.7	21.7°S, 179.5°W	580	8/3, D-16	720
8	2002-09-09	7.6	3.2°S, 142.9°E	33	9/1, D-8	770
9	2002-10-10	7.6	1.7°S, 134.2°E	10	10/4, D-6	1050
10	2002-11-02	7.4	3.0°N, 96.2°E	33	10/19, D-14	1000
11	2002-11-04	7.9	63.5°N, 147.5°W	5.0	11/3, D-1	1400
12	2002-11-17	7.3	47.9°N, 146.3°E	507.2	11/14, D-3	2750
13	2003-01-20	7.3	10.4°S, 160.8°E	33	1/12, D-8	1093
14	2003-01-22	7.6	18.8°N, 103.8°W	24	1/12, D-10	1093
15	2003-03-18	7.1	51.4°N, 177.9°E	33	3/1, D-17	1700
16	2003-05-26	7.0	38.9°N, 141.5°E	68.2	5/11, D-15	1600
17	2003-05-27	7.0	2.4°N, 128.8°E	33	5/11, D-16	1600
18	2003-06-20	7.1	7.5°S, 71.6°W	555.8	6/7, D-13	900
19	2003-07-16	7.6	2.7°S, 68.3°E	10	7/2, D-14	1000
20	2003-08-04	7.6	60.6°S, 43.5°W	10	None	
21	2003-08-21	7.2	45.1°S, 167.2°E	28	None	
22	2003-09-26	8.3	41.8°N, 143.9°E	27	9/25, D-1	1050
23	2003-09-27	7.3	49.9°N, 87.8°E	18	9/25, D-2	1050
24	2003-10-31	7.0	37.8°N, 142.6°E	10	10/27, D-4	1500
25	2003-11-17	7.8	51.1°N, 178.7°E	33	10/27, D-21	1500
26	2003-12-28	7.3	22.0°S, 169.7°E	10	12/14, D-14	1150
27	2004-02-06	7.0	3.6°S, 135.5°E	16	1/29, D-8	1700
28	2004-02-07	7.3	4.0°S, 135.0°E	10	1/29, D-9	1700
29	2004-07-25	7.3	2.5°S, 103.9°E	576	7/4, D-21	750
30	2004-09-05	7.2	33.1°N, 136.6°E	14	None	
31	2004-10-10	7.0	11.4°N, 86.7°W	35	9/21, D-19	960
32	2004-11-12	7.5	8.2°S, 124.9°E	10	10/23, D-20	1000
33	2004-11-15	7.2	4.7°N, 77.5°W	15	11/13, D-2	1300
34	2004-11-23	7.1	46.7°S, 164.8°E	10	11/13, D-10	1300
35	2004-11-26	7.1	3.6°S, 135.3°E	10	11/13, D-13	1300

36	2004-11-29	7.0	42.9°N, 145.1°E	39	11/13, D-16	1300
37	2004-12-23	8.1	50.1°S, 160.4°E	10	12/19, D-4	2100
38	2004-12-26	9.1	3.3°N, 95.9°E	30	12/19, D-7	2100
39	2005-02-05	7.1	5.4°N, 123.2°E	501.3	1/27, D-9	1050
40	2005-03-02	7.1	6.5°S, 129.9°E	201	3/1, D-1	1200
41	2005-03-29	8.6	2.1°N, 97.0°E	30	3/25, D-4	1530
42	2005-06-14	7.8	19.9°S, 69.0°W	117.2	6/13, D-1	1650
43	2005-06-15	7.2	41.3°N, 125.9°W	10	6/13, D-2	1650
44	2005-07-24	7.2	7.9°N, 92.1°E	16	None	
45	2005-08-16	7.2	38.3°N, 142.1°E	36	None	
46	2005-09-09	7.7	4.5°S, 153.4°E	91.3	None	
47	2005-09-26	7.5	5.7°S, 76.4°W	127.4	None	
48	2005-10-08	7.6	34.5°N, 73.6°E	26	10/7, D-1	1350
49	2005-11-15	7.0	38.1°N, 144.9°E	11	11/10, D-5	1650
50	2006-01-02	7.4	60.8°S, 21.5°W	10	1/2, 09:55	1300
51	2006-01-28	7.6	5.5°S, 128.1°E	397	1/12, D-16	1420
52	2006-02-23	7.0	21.3°S, 33.5°E	11	2/21, D-2	1500
53	2006-04-21	7.6	61.1°N, 167.1°E	22	4/18, D-3	1600
54	2006-05-03	8.0	20.1°S, 174.2°W	55	4/29, D-4	1870
55	2006-05-16	7.4	31.5°S, 179.3°W	151.6	5/12, D-4	1300
56	2006-07-17	7.7	9.2°S, 107.3°E	34	None	
57	2006-08-20	7.0	61.0°S, 34.4°W	10	None	
58	2006-11-15	8.3	46.6°N, 153.2°E	30.3	11/9, D-6	1100
59	2006-12-26	7.1	21.8°N, 120.5°E	10	12/14, D-12	1160
60	2007-01-13	8.1	46.3°N, 154.5°E	10	1/5, D-8	1350
61	2007-01-21	7.5	1.2°N, 126.4°E	22	1/5, D-16	1350
62	2007-03-25	7.1	20.7°S, 169.4°E	35	3/23, D-2	2700
63	2007-04-02	8.1	8.5°S, 156.9°E	10	4/1, D-1	1650
64	2007-08-02	7.2	15.7°S, 167.7°E	120	7/8, D-25	1250
65	2007-08-09	7.5	5.9°S, 107.7°E	289.2	8/4, D-5	830
66	2007-08-16	8.0	13.4°S, 76.6°W	39	8/10, D-6	750
67	2007-09-02	7.2	11.5°S, 165.8°E	35	8/26, D-7	800
68	2007-09-12	8.5	4.5°S, 101.4°E	34	9/2, D-10	1250
69	2007-09-13	7.9	2.5°S, 100.9°E	30	9/2, D-11	1250
70	2007-09-28	7.5	21.9°N, 142.7°E	261.3	9/27, D-1	1800
71	2007-09-30	7.4	49.3°S, 164.1°E	10	9/28, D-2	1670
72	2007-10-31	7.2	18.9°N, 145.3°E	248.3	10/30, D-1	2000
73	2007-11-14	7.7	22.2°S, 69.9°W	40	11/6, D-8	1000
74	2007-11-30	7.4	14.9°N, 61.2°W	146.2	11/25, D-5	2000
75	2007-12-09	7.8	25.9°S, 177.5°W	149.2	11/25, D-14	2000

76	2007-12-19	7.2	51.5°N, 179.5°E	56.3	12/17, D-2	1660
77	2008-02-20	7.4	2.8°N, 95.9°E	35	1/27, D-24	1200
78	2008-02-25	7.2	2.4°S, 100.0°E	35	2/23, D-2	1000
79	2008-03-21	7.2	35.4°N, 81.4°E	22.9	3/14, D-7	1450
80	2008-04-09	7.3	20.1°S, 168.9°E	33	4/1, D-8	1440
81	2008-04-12	7.1	55.7°S, 158.5°E	10	4/1, D-11	1440
82	2008-05-12	8.0	30.9°N, 103.4°E	19	5/2, D-10	3300
83	2008-06-30	7.0	58.2°S, 22.0°W	10	6/16, D-14	1250
84	2008-07-05	7.7	53.9°N, 152.9°E	635.6	6/16, D-19	1250
85	2008-07-19	7.0	37.6°N, 142.2°E	22	7/12, D-7	1600
86	2008-09-29	7.0	29.7°S, 177.7°W	36	None	
87	2008-11-17	7.4	1.3°N, 122.1°E	30	None	
88	2008-11-24	7.3	54.2°N, 154.3°E	491.6	11/20, D-4	1800
89	2009-01-04	7.6	0.408°S, 132.886°E	17	12/24, D-11	1100
90	2009-01-16	7.4	46.862°N,155.156°E	36	12/24, D-23	1100
91	2009-02-12	7.2	3.902°N, 126.400°E	20	2/10, D-2	1500
92	2009-03-20	7.6	23.050°S,174.668°W	34	3/16, D-4	1500
93	2009-05-28	7.3	16.733°N, 86.220°W	10	5/19, D-9	1050
94	2009-07-15	7.8	45.750°S, 166.577°E	12	7/6, D-9	900
95	2009-08-09	7.1	33.122°N,138.026°E	297	7/23, D-17	1700
96	2009-08-11	7.5	14.013°N, 92.923°E	33.1	7/23, D-19	1700
97	2009-09-02	7.0	7.783°S, 107.285°E	48.1	8/30, D-3	700
98	2009-09-30	8.0	15.509°S,172.034°W	18	9/20, D-10	1080
99	2009-09-30	7.6	0.725°S, 99.856°E	81	9/20, D-10	1080
100	2009-10-08	7.6	13.052°S, 166.187°E	35	9/20, D-18	1080
101	2009-11-09	7.3	17.21°S, 178.411°E	58	11/7, D-2	1250
