# Supplementary material for: Study on the characteristic mechanisms of infrasonic precursors during the damage process of impending earthquake sources
Source: PLoS One. 2021 Oct 1;16(10):e0257345. doi: 10.1371/journal.pone.0257345 (PMC8486096; doi:10.1371/journal.pone.0257345)
Supplement: S8 File — (DOCX) [file pone.0257345.s008.docx]

**S8. File. Characteristics of the acoustic emission pulses (total number, N) near the critical stress state (the point represents the experimental value)**

In Fig. 9, the vertical axis is the logarithmic coordinate axis and the horizontal axis is the linear coordinate axis. The parameters of the straight line 1 are as follows: slope, -0.5; intersection with the longitudinal axis, 0,6000. The parameters of the straight line 2 are as follows: slope, -0.48; intersection with the longitudinal axis, 0,130.

Experimental values near line 1:

(-0.96,110), (-0.93,80), (-0.86,65), (-0.76,38), (-0.7,35), (-0.62,25), (-0.48,7) , (-0.4,3) , (-0.32,2) , (-0.22,1.5), (-0.04,1.2)

Experimental values near line 2:

(-0.91,4100), (-0.82,1800), (-0.74,1000), (-0.61,500), (-0.5,300), (-0.44,200), (-0.4,160) , (-0.27,98) , (-0.12,30) , (-0.04,23)
